# Supplementary material for: A two-dimensional Dirac fermion microscope
Source: Nat Commun. 2017 Jun 9;8:15783. doi: 10.1038/ncomms15783 (PMC5472770; doi:10.1038/ncomms15783)
Supplement: Supplementary Information — Supplementary Figures, Supplementary Notes and Supplementary References [file ncomms15783-s1.pdf]

Supplementary Note 1 – Beam characteristics for 3 different microscope configurations.

In SI Fig. 1a-c, the three DFM configurations from Fig 3 in the main text are compared. The panel (a) shows the current density for configuration a conventional Veselago lens arrangement for five different magnetic fields (0, ± 7.5 mT and ± 15 mT). Despite the wide injection distribution from the injection contact, the Veselago lensing results in strong caustics at the backplane electrode (which is also the focal plane), even at non-zero magnetic fields. The beam profile in (d), however, is increasingly asymmetric and distorted at high magnetic fields. The angular spread for trajectories exiting at the back electrode is nearly 1 radian for all B-fields, see panel (g). Clearly, even without collimative filtering, the Veselago lens can create a narrow, focused beam of electrons, with some broadening at non-zero magnetic fields, but also with very short focal depth. Despite the asymmetric beam profile, the position of the maximal beam intensity is nearly perfectly linear in magnetic field, see panel (j).

In SI Fig. 1b and 1c, an aperture is used to limit the beam divergence. In (b) a Veselago lens halfway from the source to the backplane, focus the beam, while in (c) there is none. For both (b) and (c), configurations with and without parabolic lenses were investigated, marked in panel (d-l) as red curves (aperture), and blue curves (aperture + parabolic lens).

Panels (d-f) show the distribution of exit positions, which gives an indication of the beam shape and diameter. Panels (g-i) show the exit angle as a function of position. Panels (j-h) show the beam position as well as the beam diameter, as a function of magnetic field, however, with the axes switched to be compatible with the other panels, and shows how linearly the position depends on magnetic field.

The results differ slightly, with the sharpest beams obtained for configuration (b) without parabolic lens and configuration (c) with a parabolic lens, as in (b) the parabolic lens interferes with the focusing effect of the Veselago lens. Overall, the parabolic lens provides instead of strong focusing, a parallel, collimated beam. For configuration (c) the divergence is strongly reduced, see panel (f) and (i). In configuration (b), a small spread in angle (red) is changed to a spread in position (blue), see panel h and inset in panel (h). Both configuration (b) and (c) are close to having a linear dependence between magnetic field and position.

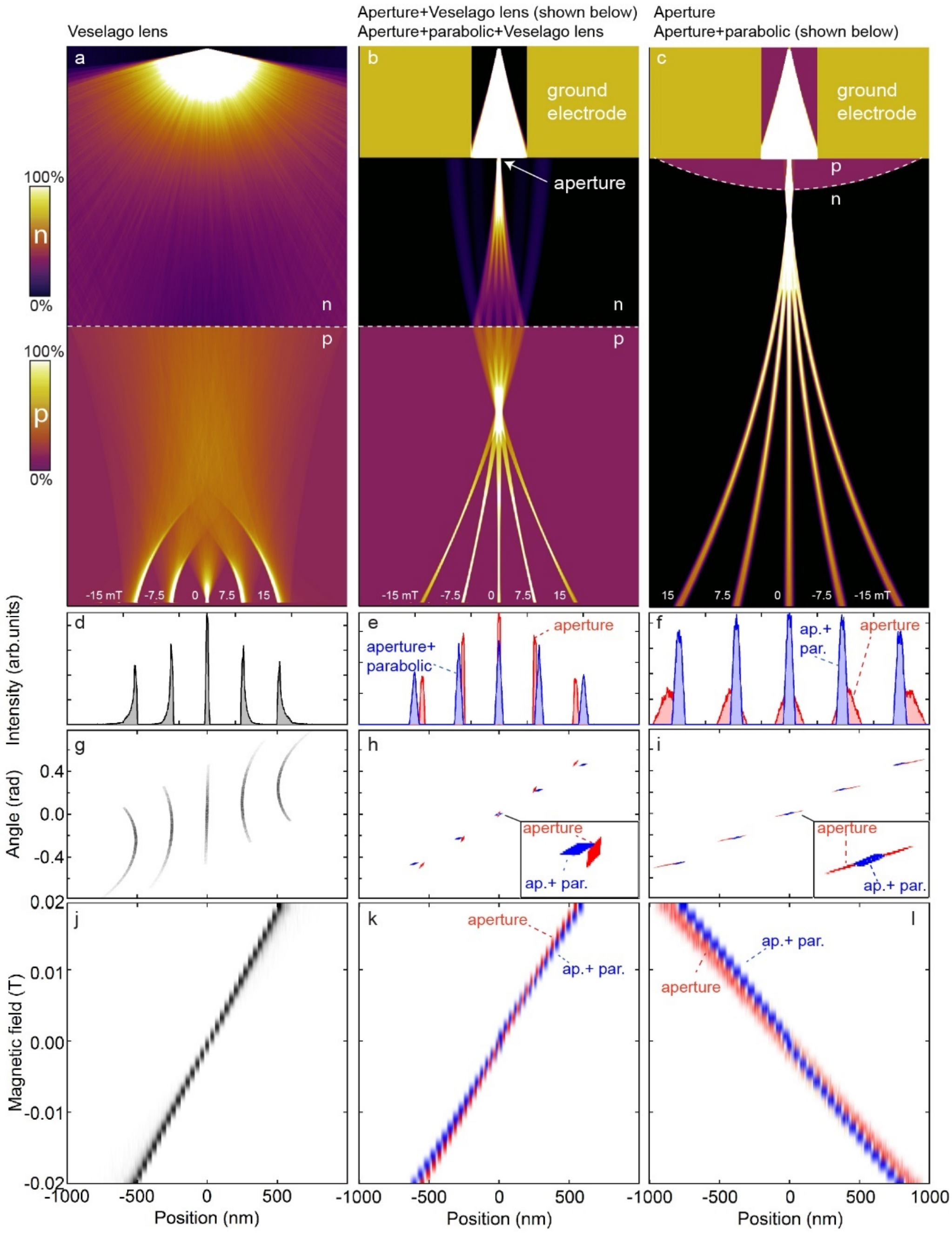

Supplementary Figure 1. Beam characteristics for 5 different microscope configurations. Trajectory current density, and beam profile, angular distribution and position linearity as a function of magnetic field for the five configurations: Veselago (**a**), Aperture/Veselago (**b**), Aperture/Parabolic lens/Veselago (not shown), Aperture (not shown), Aperture/Parabolic (**c**). (**d-f**) Distribution of exit positions at back electrode. In (**e**) and (**f**) angular distributions for aperture without (blue) and with (red) a parabolic pn-junction after the aperture are shown. (**g-i**) Angle vs position of exiting trajectories at back electrode. (**j-l**) Position vs angle for different configurations with (blue) and without (red) a parabolic lens as a function of magnetic field.

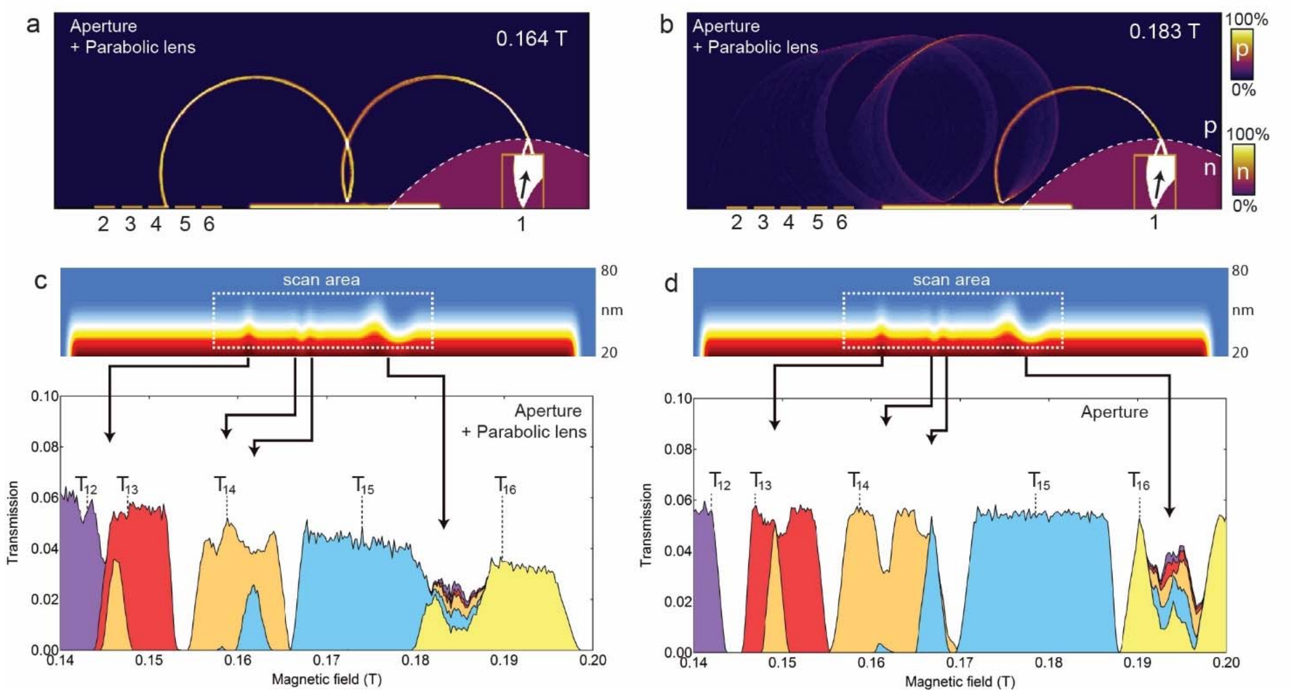

Supplementary Figure 2. Imaging roughness of edges by multiterminal magnetic focusing. An array of 5 collector electrodes can give information on the local specularity of a scattering edge. (**a**) and (**b**) show a configuration with an aperture and a parabolic lens, where the beam is scanning across a surface with roughness variations of up to 20 nm in amplitude. The magnetic focusing trajectories are seen to be nearly free from divergence in this semiclassical limit. (**c**) Image of edge roughness where the envelope of the curves is the total (accumulated) transmission, $T_{tot} = T_{12} + T_{13} + T_{14} + T_{15} + T_{16}$, and the individual contributions are shown as different colors indicated on the graphs. Local edge roughness show up as increased transmission to neighbouring detectors, while specular scattering leads to only one transmission coefficient being non-zero at a time. The largest mixing of transmission currents occurs when the magnetic focusing beam pass by the largest protrusion, which is located at around 0.183 T) for the aperture/parabolic lens configuration and at around 0.195 T for the aperture configuration without the parabolic lens. The current is more constant and the features more distinct for the gun without parabolic lens. In SI Movie 4, the current density map evolution for configuration (**b**) is shown for apertures 25 nm and 50 nm. (**d**) Image of edge roughness, using an aperture without a parabolic lens. While the image is qualitatively similar to (**c**) the total (accumulated) transmission is more constant, with a larger impact on the transmission values ($B = 0.195$ T), compared to (**b**).

Supplementary Note 2. Caustics in circular scatterers

Circular potential barriers of different sizes in graphene are well-studied scatterering objects in literature not only analytically but also experimentally. On one side, depending on the ratio between their radius and Fermi wavelength, they exhibit a diverse range of exotic transport phenomena, such as resonant scattering, quasi-bound states, caustics, rainbow and critical scattering effects[1-5]. In addition, as a first approximation circular dots and voids have been used to model scattering in graphene caused by impurities, point defects, or metallic clusters placed on the graphene sheet [6-10].

As explained in the main text, the semiclassical Monte Carlo simulations operates in the electron-optics regime, where the size of the scatterers is large in comparison with the Fermi wavelength[1]. Therefore, the evaluation of our simulations will be based on circular potentials with large size ratio, $\lambda_{\mathrm{F}} \ll R$. In particular, we perform direct comparisons between electron motion simulations obtained from our simulation and analytical approximations calculated in this optical regime for the electron motion inside circular potentials[1-3,6,8].

By considering the elastic scattering on plane electron waves in the low energy approximation, Cserti et al [1] show how the intensity maximum in graphene wavefunctions inside circular potentials form caustics which can be interpreted in the framework of geometrical optics using a negative refractive index, $n = -(k_{\mathrm{in}} / k_{\mathrm{out}}) = \sin\alpha / \sin\beta$ where k_{in} and k_{out} are the wavevectors inside and outside the circular potential (positive values)[1] and α, β are the angle of incidence and refraction, respectively (see SI Fig. 3, Inset). Here we show how the maximum current density calculated via our simulation perfectly follows these caustics, too. By doing so we demonstrate how our developed simulator is able to reproduce the complex interference patterns existing in these circular potentials, thus, ultimately checking its validity in a practical case which has been well-examined theoretically[1-3,6] and experimentally[4].

The envelope of the family of curves classified by impact parameter $b = R \sin\alpha$, where $-R \le b \le R$ (R is the radius of the circular potential) results in a caustic for each number of chords p inside the circle (corresponding to p-1 internal reflections). The curves of the caustic of the pth cord are then given in Cartesian coordinates $\begin{pmatrix} x_c(p,\alpha) \\ y_c(p,\alpha) \end{pmatrix}$, depending on the parameter α $\left(-\frac{\pi}{2} \le \alpha \le \frac{\pi}{2}\right)$

by Ref [1]:

$$\begin{pmatrix} x_c(p,\alpha) \\ y_c(p,\alpha) \end{pmatrix} = R(-1)^{p-1}\left[\begin{pmatrix} -\cos\theta \\ \sin\theta \end{pmatrix} + \cos\beta \frac{1+2(p-1)\beta'}{1+(2p-1)\beta'}\begin{pmatrix} \cos(\theta+\beta) \\ -\sin(\theta+\beta) \end{pmatrix}\right], \quad (1)$$

with $\theta(p,\alpha) = \alpha + 2(p-1)\beta$ and $\beta' = \frac{\cos\alpha}{\sqrt{n^2 - (\sin\alpha)^2}}$.

SI Figure 5 shows the corresponding caustics for the three first chords (p =1,2,3) for the case of $n = -1$, assuming $R = 1$. Along these caustics, the intensity maximum is located at a point called cusp which can be extracted by setting $\alpha = 0$ in Eq. 1. Importantly, it can be seen (SI Fig 3b) the formation of these caustics and cusps inside the circular dot is perfectly reflected in our montecarlo DMS when a circular scatterer is simultaneously impacted by a parallel beam of electrons, see Figure 5c, main text. We note how the caustics formed for larger chords p >3, are less observable since in each internal reflection the intensity of the rays is decreased.

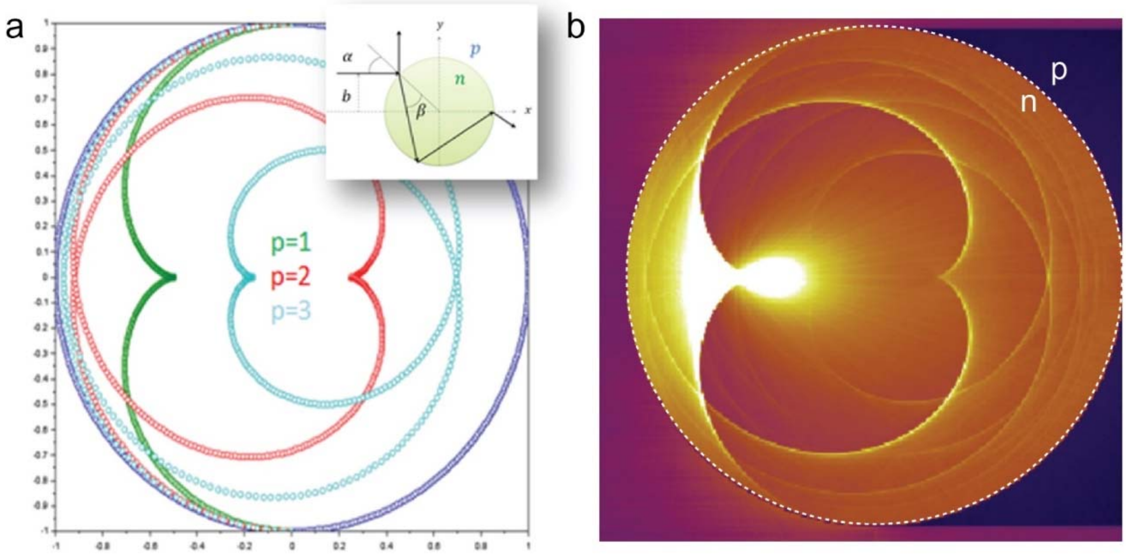

Supplementary Figure 3. Caustics inside circular scatterers due to negative refraction index. (**a**) Caustics formed for the three first cords, p =1,2,3 for an unit circle in the case of n = - 1. Inset shows the ray path inside a circular pn -junction for an impact parameter b . (**b**) Current density calculated from Monte Carlo simulation with a parallel beam of electrons a symmetric, circular pn-junction with smoothness $w = 2.5$ nm . The maximum current densities perfectly follow the caustics from SI Eq. 1, in support of the usage of our simulations in the λ_F << R regime.

Supplementary note 3. Atomistic quantum transport calculations.

We perform quantum transport simulations based on the non-equilibrium Green's function formalism within a nearest-neighbour tight-binding model, as implemented in the software package TBtrans[11]. The SISL toolbox[12] has been used to set up a two-probe device Hamiltonian where a 2 nm wide zigzag graphene nanoribbon acts as a source contact at the edge of a 100 nm$\times$100 nm graphene flake (395.940 sites), with carbon-carbon bond length $a_0 = 0.142$ nm and hopping parameter $t_0 = 2.7$ eV. A drain electrode is placed on the graphene edge opposite of the source, while all other graphene edges are equipped with a complex absorbing potential[13] to prevent artificial reflections. To ensure isotropic injection from the source we place an absorptive aperture[14] with an opening of 1.5 nm at a distance of 3 nm from the ribbon/flake interface, which here plays the role of an isotropic point source. A symmetric p-n junction with a width of $w = 2$ nm is introduced by gradually modifying the on-site terms of the tight-binding. As in Liu et al. [15], a parabolic junction with focal point located at the pinhole is used to collimate the electron beam from the source. A transverse magnetic field is accounted for using the Peierls substitution[16,17].

In order to study electron transport in our device we calculate bond-currents at $E = E_F$.

Instead of representing the individual bond current as a vector we plot the segmentof length $a_0 / 2$ corresponding to the positive bond current only and with segment thickness and color scaled according to its magnitude The same color range is used for all simulations, however, the maximum values have been adjusted for contrast. Areas with low to zero bond current appear white rather than dark, because the bond width is reduced to zero.

The area available for the quantum simulations is smaller than many of the structures considered in the main text. This can to some extent be dealt with by increasing the energy and using the scaling approximation valid for slowly varying potentials[18 19].

The bond current pattern for a small diameter d at a high energy, resembles the bond current pattern of a larger structure at low energy, provided they have similar ratio d / λ_F of diameter d and Fermi wavelength λ_F. For instance, the $d = 700$ nm diameter VD-potential as shown in Fig. 5(d) and Fig(e), will have $d \approx 20\lambda_F$ at $n = 10^{12}\,\mathrm{cm}^{-2}$, which corresponds to a 75 nm diameter VD

with $\lambda_F = 3.75$ nm and a Fermi energy of 1.1 eV. The 75 nm VD can be said to have a rescaled diameter of $d_{\text{scaled}} = 20 \times 35.4 \text{ nm} = 708 \text{ nm}$, i.e. equivalent diameter at $n = 10^{12}\ \text{cm}^{-2}$.

SI Figure 4a and 4b show the caustic bond current patterns for two VD with (a) diameter d=50 nm and (b) d=40 nm, and E = 0.4 eV and 0.5 eV, respectively, both with $d / \lambda_F = 4.9$, corresponding to the rescaled diameter $d_{\text{scaled}} = 178$ nm. As seen, the bond current patterns are virtually indistinguishable, which supports rescaling the calculations in this way, to compare with the semiclassical calculations in the main text.

SI Figure 4c-e show the caustic patterns at different energy, 1.0, 0.6 and 0.2 eV, , which corresponds to the rescaled diameters $d_{\text{scaled}} = 428$ nm, 257 nm and 86 nm, respectively. The caustic pattern from SI Fig. 3 superposed on SI Fig. 4d, are in reasonable agreement and very similar to the caustic patterns shown in Fig. 1 in Agrawal et al [20].

SI Figure 4f-h shows a focused, collimated electron beam scattering on a 75 nm diameter circular pn-junction for different energies around 1 eV. The rescaled diameters $d_{\min}$ are in the range 707 nm to 578 nm. SI Figure 4i is 60 nm in diameter and an energy of $E_F = 0.9$ eV, which results in a $d_{\text{scaled}} = 578$ nm. The d / λ_F ratio is nearly the same, 16.0 and 16.4, in the two cases, (h) and (i), and the bond current patterns are indeed very similar. The SI Fig. 4f has the same scaled diameter $d_{\text{scaled}} = 707$ nm as the VD simulated in Fig. 5e (main text), and similar current patterns both with respect to emission jets and the internal, triangularly shaped current structure. SI Fig. 4j-m show the bond currents for different magnetic fields, which are in rough qualitative agreement with Fig. 3 and Fig. 5 in the main text.

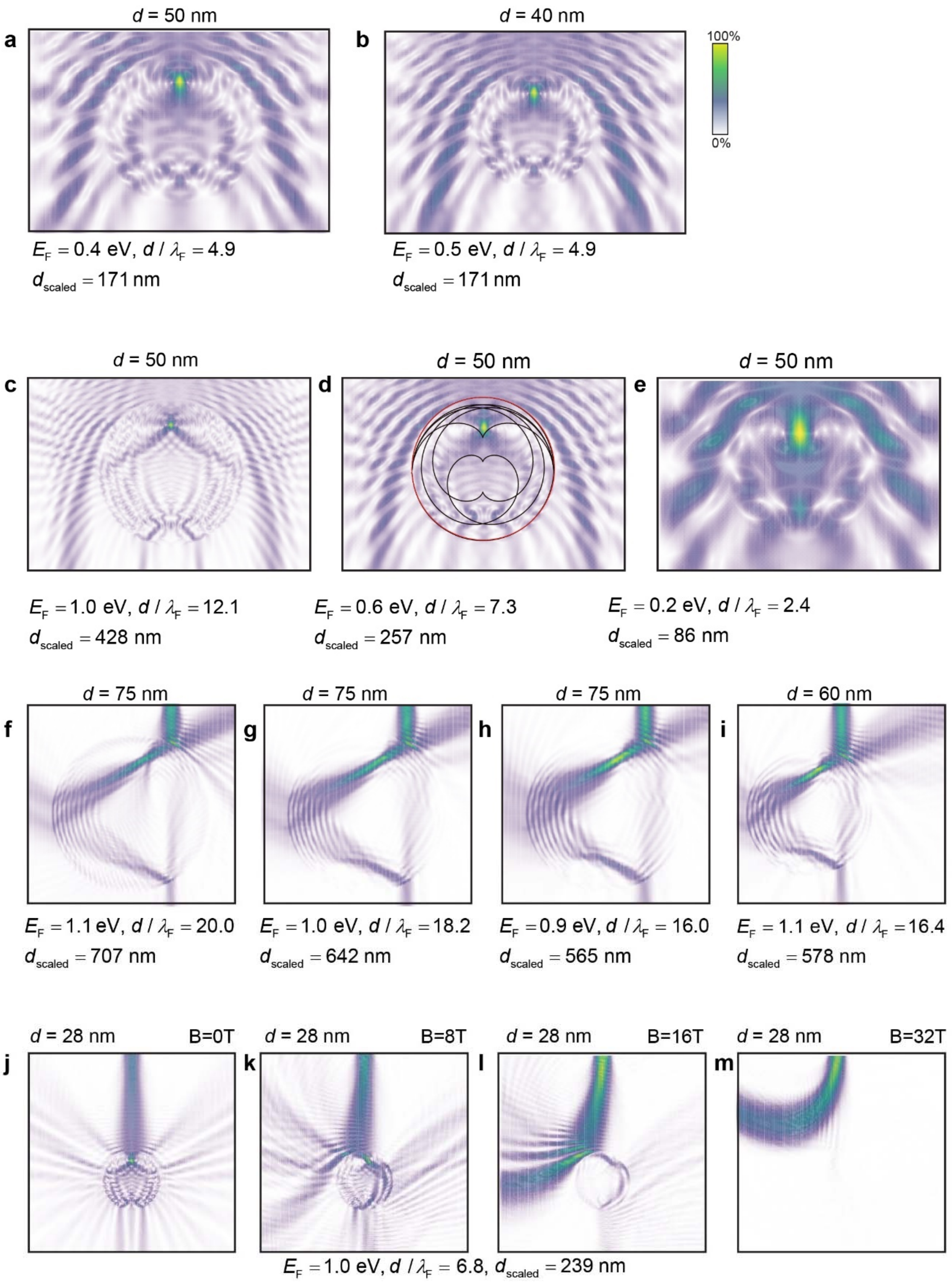

Supplementary Figure 4. Quantum transport calculations of DFM scattering from circular VD. (**a-b**) Comparison of bond current caustic pattern of two VD with different size and energy, but same $d/\lambda_F = 4.9$. (**c-e**) Bond current caustic patterns for a 50 nm VD with $E_F = 1.0$ eV, 0.6 eV and 0.2 eV . These energies correspond to VDs with diameters $d_{scaled} = 428$ nm,

257 nm and 86 nm for $n = 10^{12}\,\text{cm}^{-2}$ ($E_\text{F} = 0.117$ eV), used in the main text. The classical caustics from SI Fig. 3 are superposed on panel (**d**). (**f-h**) A focused DF beam is impinging on a large circular VD with a diameter of 75 nm and energies $E_\text{F} = 1.1$ eV, 1.0 eV and 0.9 eV. The bond current distribution for E=1.1 eV ($d_\text{scaled} = 707$ nm) is in agreement with the semiclassical current density of the 700 nm dot diameter in Fig 5h, main text. Comparison of (**h**) and (**i**) confirms that structures with similar $d / \lambda_\text{F} \approx 16$ have very similar bond current distribution. (**j-m**) Collimated DF beam scanning across a small VD ($d = 28$ nm, $\text{d}_\text{scaled} = 239$ nm) with an energy $E_\text{F} = 1.0$ eV. The bond current scattering patterns roughly resemble the semiclassical simulations shown in Fig. 4, and Fig. 6, main text.

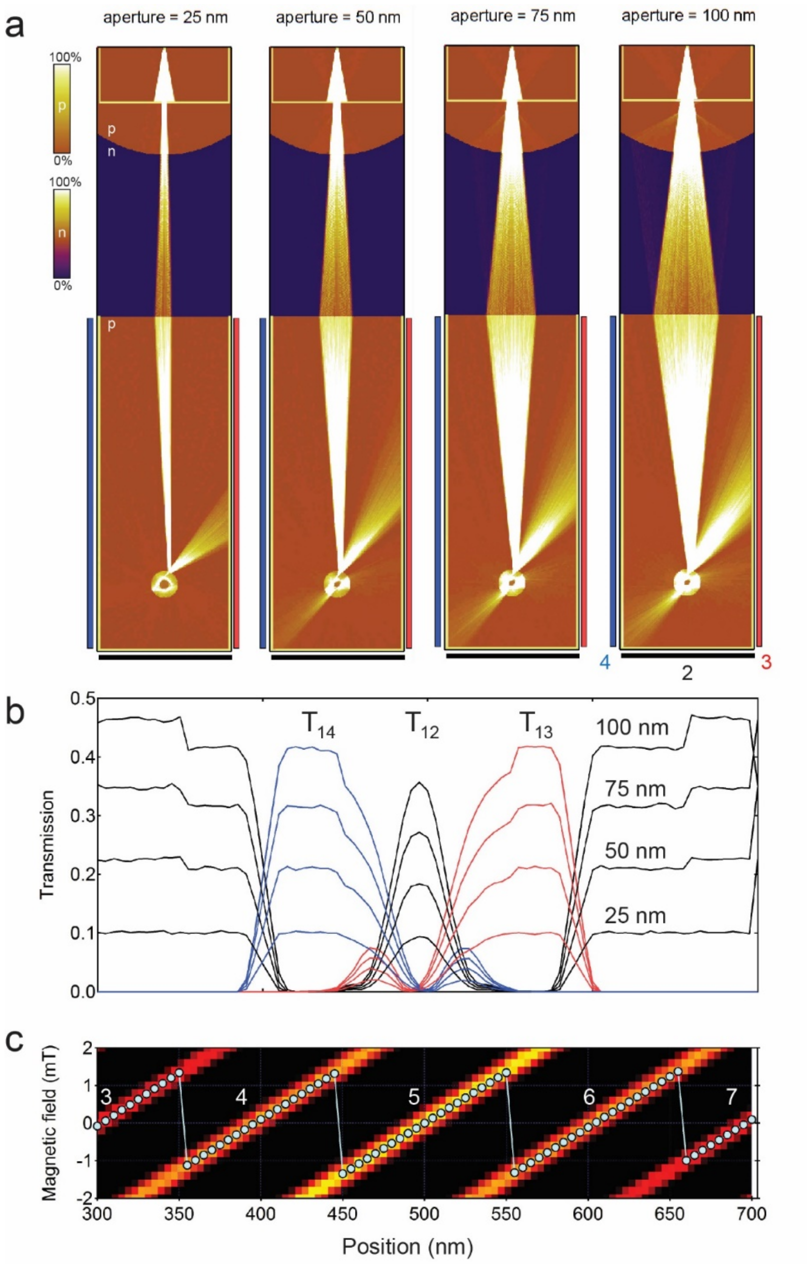

Supplementary Figure 5. DFM with increasing aperture. (**a**) Current density plots for the aperture being increased in steps of 25 nm from 25 nm to 100 nm, with a 200 nm diameter circular pn-junction (smoothness w = 10 nm) as the target. (**b**) The transmission images at the three electrodes 2, 3 and 4 are similar except for a scaling factor due to the higher currents passing through the larger aperture. The current stitching errors are increasing (i.e. at 350 nm and 660 nm) as the current calibration for the smallest aperture was used for all four curves. (**c**) The magnetic field and emitters are shown versus beam position to illustrate the stitching scheme, with the changes from emitter 3 through 7.

Supplementary Note 4. Suggested process flow for DFM.

We provide here one possible solution to fabricate the Dirac fermion microscope (DFM) configuration with the coaligned beam and multiple emitters, which is the most complex of our devices. The process flow is a suggestion for a starting point for fabricating the conceptual device, and will most likely require further adaption and optimization. The fabrication process is based on the hot-pickup van der Waals method, Pizzocchero et al, Nat. Comm (2016), Ref. [21].

SI Figure 6 shows in panel (a) an illustration of the DFM and (b) a Monte Carlo simulation of a 4.5 μm x 1 μm DFM with 9 emitters. The 100 nm resolution requirement to define the emitter array as well as the narrow aperture can be demanding, so scaling the device by a factor of 5-10 (as discussed in the main text) will make it far easier to fabricate with standard electron beam lithography systems (typical : 30 keV), as well as reduce diffraction through the narrow aperture. SI Figure 6(c) illustrates a piece of few-layer graphite (below 10 layers) for the backgate that defines both the parabolic and the flat Veselago lens (see Fig. 5 in main text). SI Figure 6 (d) shows the graphite blockpatterned with a positive, high resolution resist (e.g PMMA, ZEP-520A or CSAR), and etched in an oxygen plasma. Four alignment marks are left to enable correct aligning of the subsequent lithography patterns (SI Fig. 6(e)). A large clean stack is prepared by self-cleaning (hot-pickup method) and dropped down on the graphite gate, shown in top view in SI Fig. 6(f). The bottom hBN should be around 10 nm or less, for the electron lenses to work optimally. SI Fig. 6(g) illustrates the second lithography step which defines the stack as well as the emitter leads. In SI Fig. 6(h) the resist is here used as an etch mask directly; alternatively, an Al etch mask can be used to achieve better etch resistance and definition of the finest structures. After etching of the three layers by a SF_6 etch (hBN), a brief oxygen plasms (graphene) followed by a second SF_6 etch (hBN) as described in Ref [21], the device is ready for metal contacts. SI Fig. 6 (i) illustrates a third lithography step , followed by $SF_6/O_2/SF_6$ etch to open the stack for electrical contacts(SI Fig. 6 (j)), as well as to the graphite back gate. Cr/Au or Cr/Pd/Au contacts deposition and lift-off, concludes the process.

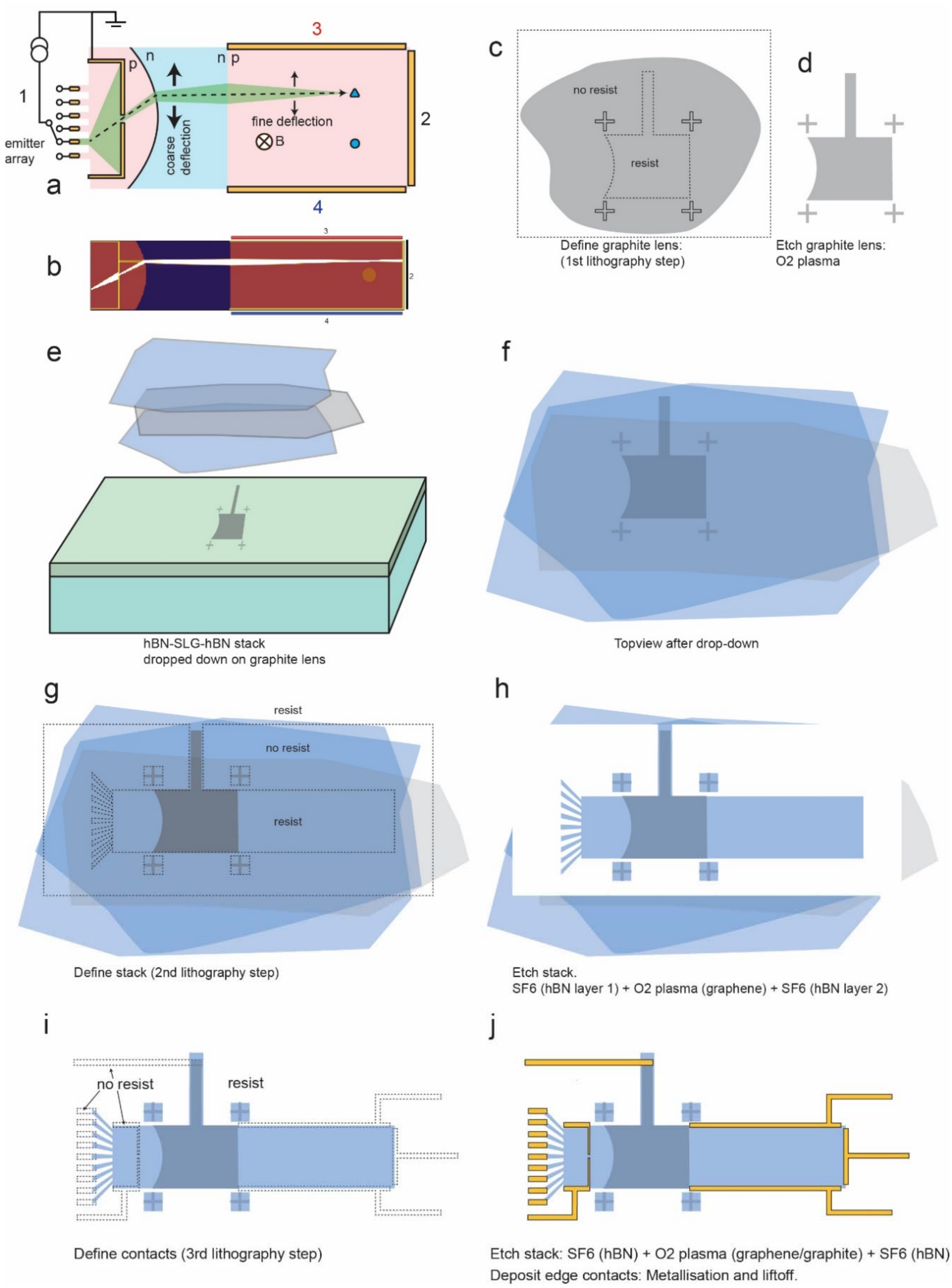

Supplementary Figure 6. Possible process flow to fabricate DFM devices. The process is based on the hot-pickup technique[21] and previous methods mentioned herein, and is intended as a starting point. See SI Note 4, for description of the individual steps.

References

1 Cserti, J., Palyi, A. & Peterfalvi, C. Caustics due to a negative refractive index in circular graphene p-n junctions. *Physical Review Letters* **99**, 246801 (2007).

2 Heinisch, R. L., Bronold, F. X. & Fehske, H. Mie scattering analog in graphene: Lensing, particle confinement, and depletion of Klein tunneling. *Physical Review B* **87**, 155409 (2013).

3 Wu, J. S. & Fogler, M. M. Scattering of two-dimensional massless Dirac electrons by a circular potential barrier. *Physical Review B* **90**, 235402 (2014).

4 Caridad, J. M., Connaughton, S., Ott, C., Weber, H. B. & Krstic, V. An electrical analogy to Mie scattering. *Nature Communications* **7**, 12894 (2016).

5 Logemann, R., Reijnders, K. J. A., Tudorovskiy, T., Katsnelson, M. I. & Yuan, S. J. Modeling Klein tunneling and caustics of electron waves in graphene. *Physical Review B* **91**, 045420 (2015).

6 Katsnelson, M. I., Guinea, F. & Geim, A. K. Scattering of electrons in graphene by clusters of impurities. *Physical Review B* **79**, 195429 (2009).

7 McCreary, K. M. *et al.* Effect of cluster formation on graphene mobility. *Physical Review B* **81**, 115453 (2010).

8 Guinea, F. Models of Electron Transport in Single Layer Graphene. *Journal of Low Temperature Physics* **153**, 359-373 (2008).

9 Hentschel, M. & Guinea, F. Orthogonality catastrophe and Kondo effect in graphene. *Physical Review B* **76**, 115407 (2007).

10 Vaishnav, J. Y., Anderson, J. Q. & Walls, J. D. Intravalley multiple scattering of quasiparticles in graphene. *Physical Review B* **83**, 165437 (2011).

11 Papior, N., Lorente, N., Frederiksen, T., Garcia, A. & Brandbyge, M. Improvements on non-equilibrium and transport Green function techniques: The next-generation TRANSIESTA. *Computer Physics Communications* **212**, 8-24 (2017).

12 Nick R. Papior. sisl: v0.8.3. (2017). doi:10.5281/zenodo.495025

13 Xie, H., Kwok, Y., Jiang, F., Zheng, X. & Chen, G. H. Complex absorbing potential based Lorentzian fitting scheme and time dependent quantum transport. *Journal of Chemical Physics* **141**, 164122 (2014).

14 Barnard, A. W. *et al.* Absorptive pinhole collimators for ballistic Dirac fermions in graphene. *Preprint at* https://arxiv.org/abs/1611.05155 (2016).

15 Liu, M.-H., Gorini, C. & Richter, K. Creating and Steering Highly Directional Electron Beams in Graphene. *Physical Review Letters* **118**, 066801 (2017).

16 Peierls, R. On the theory of diamagnetism of conduction electrons. *Zeitschrift Fur Physik* **80**, 763-791 (1933).

17 Pedersen, J. G. & Pedersen, T. G. Tight-binding study of the magneto-optical properties of gapped graphene. *Physical Review B* **84**, 115424 (2011).

18 Liu, M. H. *et al.* Scalable Tight-Binding Model for Graphene. *Physical Review Letters* **114**, 036601 (2015).

19 Beconcini, M. *et al.* Scaling approach to tight-binding transport in realistic graphene devices: The case of transverse magnetic focusing. *Physical Review B* **94**, 115441 (2016).

20 Agrawal, N., Ghosh, S. & Sharma, M. Scattering of massless Dirac fermions in circular p-n junctions with and without magnetic field. *Journal of Physics-Condensed Matter* **26**, 155301 (2014).

21 Pizzocchero, F. *et al.* The hot pick-up technique for batch assembly of van der Waals heterostructures. *Nature Communications* **7**, 11894 (2016).
